# Supplementary material for: Critical role of WNK1 in MYC-dependent early mouse thymocyte development
Source: eLife. 2020 Oct 14;9:e56934. doi: 10.7554/eLife.56934 (PMC7591260; doi:10.7554/eLife.56934)
Supplement: Transparent reporting form [file elife-56934-transrepform.docx]

***eLife’s* transparent reporting form**

We encourage authors to provide detailed information *within their submission* to facilitate the interpretation and replication of experiments. Authors can upload supporting documentation to indicate the use of appropriate reporting guidelines for health-related research (see [EQUATOR Network](http://www.equator-network.org/%20)), life science research (see the [BioSharing Information Resource](https://biosharing.org/)), or the [ARRIVE guidelines](http://www.plosbiology.org/article/info:doi/10.1371/journal.pbio.1000412) for reporting work involving animal research. Where applicable, authors should refer to any relevant reporting standards documents in this form.

If you have any questions, please consult our Journal Policies and/or contact us: [editorial@elifesciences.org](mailto:editorial@elifesciences.org).

**Sample-size estimation**

- You should state whether an appropriate sample size was computed when the study was being designed
- You should state the statistical method of sample size computation and any required assumptions
- If no explicit power analysis was used, you should describe how you decided what sample (replicate) size (number) to use

Choice of sample size was based on the variance seen in previous experiments using the same or similar experimental approaches.

Please outline where this information can be found within the submission (e.g., sections or figure legends), or explain why this information doesn’t apply to your submission:

**Replicates**

- You should report how often each experiment was performed
- You should include a definition of biological versus technical replication
- The data obtained should be provided and sufficient information should be provided to indicate the number of independent biological and/or technical replicates
- If you encountered any outliers, you should describe how these were handled
- Criteria for exclusion/inclusion of data should be clearly stated
- High-throughput sequence data should be uploaded before submission, with a private link for reviewers provided (these are available from both GEO and ArrayExpress)

The number of times experiments were carried out and the number of samples (n numbers) for each experiment can be found directly in the figure legends. N numbers always refer to biological repeats, not to technical repeats. Biological repeats were defined as analysis of different animals, or of cells from different animals. Repeat measurements on cells from the same animal were defined as technical repeats. Only biological repeats were used for statistical analysis. No outliers were removed and no experiments were excluded. The RNAseq data has been uploaded to GEO. The accession number and the token required for reviewers to access the data can be found in the Methods section.

Please outline where this information can be found within the submission (e.g., sections or figure legends), or explain why this information doesn’t apply to your submission:

**Statistical reporting**

- Statistical analysis methods should be described and justified
- Raw data should be presented in figures whenever informative to do so (typically when N per group is less than 10)
- For each experiment, you should identify the statistical tests used, exact values of N, definitions of center, methods of multiple test correction, and dispersion and precision measures (e.g., mean, median, SD, SEM, confidence intervals; and, for the major substantive results, a measure of effect size (e.g., Pearson's r, Cohen's d)
- Report exact p-values wherever possible alongside the summary statistics and 95% confidence intervals. These should be reported for all key questions and not only when the p-value is less than 0.05.

Please outline where this information can be found within the submission (e.g., sections or figure legends), or explain why this information doesn’t apply to your submission:

The statistical tests used for analyzing the data, n-numbers, repeats and precision measures (mean±SEM) are described in the Figure Legends and in the Methods. Whenever possible examples of the raw data are provided directly in the figures, this includes flow cytometry plots with gating strategies, histograms showing protein expression (e.g. intracellular TCRbeta, MYC expression, amount of DNA), immunoblots showing levels of OXSR1 phosphorylation, microscopy images. Raw data for the RNAseq experiment has been uploaded to GEO, while analyzed RNAseq data can be found in the Supplementary Tables showing differential gene expression analysis with associated adjusted p-values. On the Figures, p-values are indicated as ranges, e.g. * 0.01 < p < 0.05.

(For large datasets, or papers with a very large number of statistical tests, you may upload a single table file with tests, Ns, etc., with reference to sections in the manuscript.)

**Group allocation**

- Indicate how samples were allocated into experimental groups (in the case of clinical studies, please specify allocation to treatment method); if randomization was used, please also state if restricted randomization was applied
- Indicate if masking was used during group allocation, data collection and/or data analysis

Please outline where this information can be found within the submission (e.g., sections or figure legends), or explain why this information doesn’t apply to your submission:

No randomization of animals was done. No masking or blinding was used. For each experiment all samples were always analyzed with the same criteria, e.g. using identical gating strategies for flow cytometry experiments.

**Additional data files (“source data”)**

- We encourage you to upload relevant additional data files, such as numerical data that are represented as a graph in a figure, or as a summary table
- Where provided, these should be in the most useful format, and they can be uploaded as “Source data” files linked to a main figure or table
- Include model definition files including the full list of parameters used
- Include code used for data analysis (e.g., R, MatLab)
- Avoid stating that data files are “available upon request”

Please indicate the figures or tables for which source data files have been provided:

The RNAseq data that was used for the analysis shown in Figures 3D,E, 4A-C, 5A, 6A, and 7A,B, Figure 4-Figure supplement 1A, Figure 6-Figure supplement 1, Figure 7-Figure supplement 1A can be found in Supplementary Tables 1 to 3.
